# Periapical Status of Post-Restored Teeth: A Two-Year Follow-Up Study

**DOI:** 10.3390/dj14050270

**Published:** 2026-05-05

**Authors:** Chaimae Loudili, Amine Roufid, Fatima Ezzahra Faridi, Rime Chraibi, Hicham Soualhi, Babacar Toure

**Affiliations:** 1 Department of Fixed Prosthodontics, International Faculty of Dental Medicine of Rabat, International University of Rabat, Parc Technopolis Rabat-Shore, Rocade Rabat-Salé, Sala Al Jadida 11100, Morocco; rime.chraibi@uir.ac.ma; 2 Department of Conservative Dentistry and Endodontics, International Faculty of Dental Medicine of Rabat, International University of Rabat, Parc Technopolis Rabat-Shore, Rocade Rabat-Salé, Sala Al Jadida 11100, Morocco; amine.roufid@uir.ac.ma (A.R.); fatima-ezzahra.faridi@uir.ac.ma (F.E.F.); babacar.toure@uir.ac.ma (B.T.); 3 Department of Fixed Prosthodontics, Mohammed V Dental University of Rabat, Madinat Al Irfane, Rabat 6212, Morocco; soualhihicham@yahoo.fr

**Keywords:** post-restored teeth, periapical lesion, endodontic treatment, radiographic evaluation, clinical follow-up

## Abstract

**Objectives:** This study evaluated the prevalence of periapical lesions in endodontically treated teeth restored with cast post-and-core systems after a minimum two-year follow-up, and identified restorative and clinical factors influencing periapical health. **Methods:** A cross-sectional study was conducted at the dental clinic of the International University of Rabat. Of 224 contacted patients, 91 met the inclusion criteria (completed endodontic treatment and post placement > 2 years), yielding 118 post-restored teeth. Radiographs were obtained using a phosphor plate system and analyzed with DBSWIN software, applying Ørstavik’s PAI index. Statistical analysis was performed with Jamovi (v2.3.24, Australia). Quantitative data were expressed as mean and standard deviation, and qualitative data as numbers and percentages. The Chi-square test was used with a significance level of *p* < 0.05. **Results:** The study found that 31.4% of the teeth presented a radiologically detectable periapical lesion (PAI ≥ 3). Maxillary incisors and premolars were the most frequently affected. Lesion prevalence was significantly associated with post/filling gaps ≥ 2 mm (*p* = 0.008) and low-density root fillings (*p* < 0.001). Although short filling length (<4 mm) was common in teeth with periapical lesions, no significant association was observed (*p* = 0.550). Systemic conditions, such as diabetes, showed a trend toward higher lesion prevalence (*p* = 0.056). **Conclusions:** The findings highlight the need for a rigorous approach in restoring endodontically treated teeth. Absence of gaps between the root canal filling and post and homogeneous filling density are key protective factors for maintaining periapical health. Regular radiographic follow-up and future longitudinal studies (3D imaging and clinical parameters) remain essential to refine protocols and improve outcomes.

## 1. Clinical Significance

Achieving a homogeneous three-dimensional obturation and eliminating gaps between the post and the root canal filling significantly reduces the risk of periapical failure. Clinicians should verify post adaptation radiographically before cementation and prioritize high-density obturation techniques.

## 2. Introduction

Post-and-core restorations are commonly used to rehabilitate endodontically treated teeth with substantial loss of coronal structure [1]. Their long-term success depends on several factors, including the quality of the remaining tooth structure, root morphology, and the condition of periapical tissues [2,3]. Adequate root canal obturation, maintaining 4–6 mm of apical gutta-percha, is essential to prevent microleakage and preserve periapical health [4,5].

Despite their biomechanical effectiveness, root posts may also be implicated in the development or persistence of periapical lesions. The underlying mechanisms include microleakage, root fractures, perforations, or inadequate sealing, all of which may compromise the integrity of endodontic obturation [1].

Recent radiographic evidence also highlights variability in the technical quality of post-and-core restorations. Almansour et al. [6] reported that key parameters such as post length and gutta-percha homogeneity are frequently suboptimal, potentially compromising the apical seal and long-term periapical health. Importantly, Farah et al. [7] demonstrated a significant association between periapical lesions and both the quality of root canal fillings and the type of intraradicular post, reporting a prevalence of apical periodontitis of 22%, which increased to 38% in teeth with inadequate root canal fillings, compared with 14% in adequately treated roots.

A national descriptive survey conducted in Morocco in 2023 reported that failures occurred most frequently in teeth restored with post-supported crowns (50%), with endodontic failures predominating (66%) [8]. Similarly, an epidemiological study, carried out in Dakar, by Touré et al. [9] found 53% of apical periodontitis in teeth restored with fixed prostheses; results consistent with those of Saunders et al. [10], who reported 51% in root-filled teeth with fixed restorations.

To date, few studies have focused specifically on the periapical status of teeth restored with post-and-core restorations in Morocco. Therefore, the present study aims to evaluate, through periapical radiographs, the periapical condition of teeth restored with cast post-and-core restorations after at least two years at the Department of Fixed Prosthodontics, International University of Rabat. The objectives are to determine the prevalence of periapical lesions and to assess their association with the quality of root canal obturation and post placement.

## 3. Materials and Methods

### 3.1. Study Design and Setting

This retrospective observational study assessed the periapical status using the Periapical Index (PAI) on radiographs obtained at least two years after endodontic treatment and post placement, at the dental clinic of the International University of Rabat between January and June 2025.

The study protocol was approved by the Research Ethics Board of the International Faculty of Dental Medicine of Rabat-International University of Rabat (CUMD/FIMD 001/20/24/Approval/2024) for ethical approval of research involving human participants.

### 3.2. Participants

All of the 224 patients who had received post-and-core restorations in the Department of Dentistry of the University were contacted by phone to participate in the study. The inclusion criteria include:

- Patients who had undergone endodontic treatment followed by coronoradicular reconstruction using cast metal post-and-core systems, and subsequent restoration with full-coverage crowns.
- Patients who attended the recall visit and provided informed consent to participate during the study period (January to June 2025).

The Exclusion/non-inclusion criteria include:

- Teeth with recent endodontic treatments (<2 years).
- Patients who had changed residence or phone number.
- Patients who refused to attend for follow-up radiographs.
- Patients with non-metallic posts (e.g., glass-fiber-reinforced posts).
- Patients with special precautions regarding radiographs (e.g., pregnant women in the first trimester).

### 3.3. Radiographic Protocol

The radiographs were taken using phosphor plates developed by the digital Vista Scan Mini device (Dürr Dental, Bietigheim-Bissingen, Germany) available in the radiology rooms of the university clinic, known for its high resolution and reproducibility in periapical radiographs.

The images were viewed and analysed using the DBSWIN software to interpret and extract data from the digital radiographs, with zoom and contrast enhancement tools to optimize image reading.

Radiographs were taken following a standardized protocol to ensure consistency in image quality and positioning. A universal film holder with angulators was used to minimize distortions and ensure reproducibility of the images.

Two independent examiners, trained in endodontic radiographic assessment, evaluated each radiograph. In cases of disagreement, consensus was reached through discussion.

Two calibration sessions were conducted with a 2-week interval between them before beginning radiographic analysis.

At the first session, 100 radiographs chosen from a previous study (Touré et al. [11]) with different periapical status covering all the PAI scales were examined by all the examiners. Interexaminer agreement, calculated as Cohen’s kappa, ranged from 0.80 to 0.95.

At the second session, 75 different radiographs taken from the same material were examined. Intraexaminer reproducibility was evaluated with a Cohen’s kappa ranging from 0.83 to 0.92. When disagreement in interpretation was noted between observers, radiographs were re-examined until an agreement on PAI score was noted.

The radiographs included in the analysis were those obtained at the time of post placement (with a minimum follow-up of two years), as well as more recent radiographs taken during the study.

### 3.4. Variables

The data were focused on both sociodemographic and clinical variables. Sociodemographic data included the patient’s age, sex, and general health status as recorded in the medical file.

From a clinical and radiographic standpoint, the presence or absence of periapical lesions was evaluated before and after treatment. The severity of these lesions was assessed according to the Periapical Index (PAI) described by Ørstavik et al. [12], which provides a standardized scoring system ranging from 1 to 5 according to the severity of radiographic periapical changes; PAI > 2 was considered to be a sign of periapical disease.

The quality of root canal treatment was evaluated based on the density and homogeneity of the obturation material, with particular emphasis on the apical condensation. The length of the remaining apical obturation was also measured.

Finally, the presence of a gap between the post and the residual root canal filling was visually assessed on periapical radiographs and categorized as present or absent. The evaluation was performed independently by two examiners, and in cases of disagreement, a consensus meeting was held to determine the final outcome.

### 3.5. Statistical Analysis

The variables analyzed in this study fall into two categories: qualitative and quantitative. Qualitative variables were expressed as numbers and percentages, while quantitative variables were expressed as means and standard deviations.

All data were analyzed using Jamovi software (version 2.3.24, Australia).

The chi-square test (χ^2^) was used to compare variables, with the level of significance set at *p* ≤ 0.05.

## 4. Results

The study initially included 295 teeth restored with cast posts. A total of 224 patients were contacted for this study, of whom 91 attended the recall visit and were included in the final analysis. The final sample consisted of 67 women (mean age: 41.0 ± 11.2 years) and 24 men (mean age: 40.2 ± 15.5 years). The mean and median ages were similar (41.0 vs. 40.2 years), indicating a comparable age distribution between the two groups. Among men, ages ranged from 18 to 69 years, representing both the youngest and oldest participants in the sample.

Although the age distributions of men and women were broadly similar (median: 41 vs. 38.5 years), variability was greater in men (standard deviation: 15.5 vs. 11.2). Overall, these findings suggest that age is unlikely to act as a major confounding factor in this study, although the higher dispersion among men should be considered when interpreting stratified analyses.

The sample was distributed relatively evenly across the 18–30 (30.8%), 31–40 (27.5%), and 41–50 (28.6%) year age groups. As expected, individuals over 50 years of age were underrepresented (13.2%). This distribution is representative of the typical target population in conservative dentistry, while still allowing for comparative analyses across the defined age strata. One hundred and twenty-six teeth were selected for radiographic evaluation. However, 8 teeth were excluded due to their absence from the dental arch at the time of examination, which prevented a reliable radiological analysis.

Thus, 118 teeth were included in the statistical analysis. The analysis reveals a strongly asymmetric distribution of teeth between the arches. The maxilla (83.9% of teeth) shows a marked specialization for incisors (56.6% of its teeth), while the mandible (16.1%) is dominated by premolars (68.4%). This dichotomy likely reflects differential anatomical constraints, although a selection bias cannot be ruled out given the low mandibular representation (*n* = 19). The low representation of molars in both arches (6.1% maxilla, 15.8% mandible) may warrant further investigation.

In this present study, the distribution of the Ørstavik index showed that 44.1% of teeth maintained the most favorable level (1), while 24.6% exhibited mild changes (2). Only 9.3% reached a moderate level (3), whereas 21.2% developed severe alterations (4) (Table 1).

**Figure 1:**
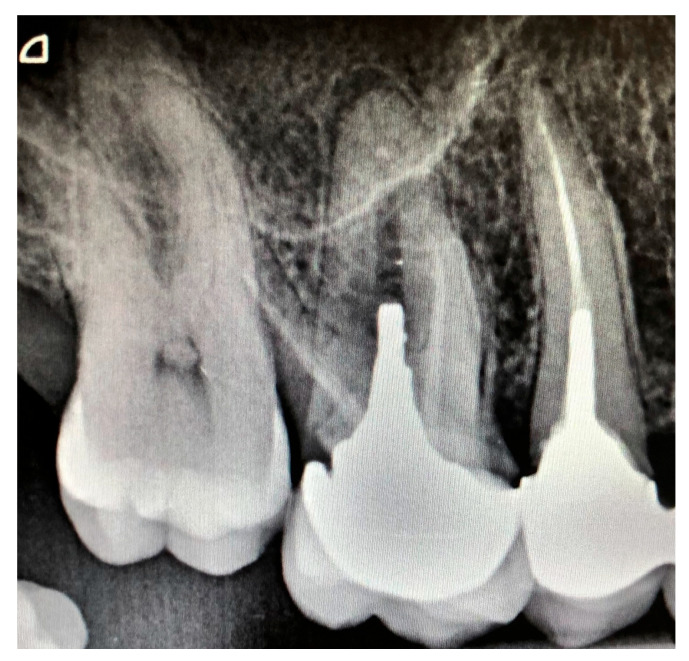
Representative periapical radiograph illustrating a PAI score of 2 on tooth 15 and a PAI score of 4 on tooth 16.

A single case (0.8%) was classified as critical (Figure 1).

No association was found between patient sex and the prevalence of periapical lesions (*p* = 0.901). The proportion of teeth with lesions was nearly identical between women (31.7%) and men (30.6%). These results indicate that sex is not a significant risk factor for periapical lesions following post-and-core restorations in this survey.

According to the length of the remaining apical filling, the (Table 2) shows the distribution of the 118 teeth restored with post-and-core, according to two main variables:

1. The length of the remaining apical seal, is classified into two categories:
  - Less than 4 mm (denoted as “no”).
  - Greater than or equal to 4 mm (denoted as “yes”).
2. The radiographic periapical status, is assessed by the presence or absence of a periapical lesion.

No statistically significant association was found between the length of the remaining apical seal and periapical status (*p* = 0.550). Although a trend was observed—with a lower prevalence of periapical lesions in teeth with an adequate seal (≥4 mm; 29.6%) compared towith those with an inadequate seal (<4 mm; 35.1%)—the difference was not significant. This lack of statistical significance may be attributable to the study’s limited sample size.

A significant association was found between the presence of a post-obturation gap and periapical health (*p* = 0.008). While 78.3% of teeth without a gap were healthy, only 55.1% of those with a gap exhibited no periapical lesion. Conversely, the prevalence of periapical lesions was more than twice as high in the gap group (44.9%) compared with the no-gap group (21.7%). These results indicate that a gap between the post and root canal filling is a significant risk factor, likely compromising the apical seal and permitting microbial leakage that leads to periapical disease. The analysis of periapical status according to root canal filling density revealed a significant influence of this parameter on the presence of apical lesions. Among the 68 teeth with radiographically inadequate filling density, 30 (44.1%) exhibited a periapical lesion, whereas 38 (55.9%) did not. In contrast, among the 50 teeth with satisfactory filling density, only 7 (14.0%) presented an apical lesion, while 43 (86.0%) appeared normal radiographically. Overall, 37 teeth (31.4%) showed evidence of periapical pathology. Statistical analysis demonstrated a highly significant difference in the prevalence of periapical lesions between the two groups (*p* < 0.001). Teeth with satisfactory filling density exhibited a markedly lower prevalence of periapical lesions compared with those with inadequate filling. These findings suggest that a dense and homogeneous root canal filling is strongly associated with improved periapical health, potentially due to reduced apical microleakage and a lower risk of persistent or recurrent infection (Table 2).

When analyzing periapical status according to the patients’ general health condition, a non-significant trend was observed. Among patients without systemic health conditions (*n* = 112), 33 teeth (29.5%) exhibited an apical radiolucency, whereas among patients with compromised general health (*n* = 6), 4 teeth (66.7%) showed a radiolucency. Although the difference did not reach the conventional level of statistical significance (*p* = 0.056), the notably higher prevalence of periapical lesions in patients with systemic health compromise suggests a potential association that warrants further investigation in larger cohorts. This trend may indicate that systemic factors could influence periapical healing or resistance to infection following endodontic treatment.

Regarding tooth type (Table 3), the prevalence of periapical lesions varied across different categories. Maxillary incisors exhibited a lesion prevalence of 32.1% (18/56), accounting for nearly half (48.6%) of all detected lesions. Maxillary premolars presented the highest lesion rate (33.3%), while mandibular premolars demonstrated the lowest prevalence (23.1%), possibly reflecting differences in bone density and anatomical characteristics between the maxilla and the mandible. Overall, the prevalence of periapical lesions across the sample was 31.4%. Since maxillary incisors represented the most frequent tooth type (47.5% of the total sample), their lesion rate was close to the overall mean. However, due to the limited sample size of certain subgroups (e.g., *n* ≤ 3 for canines and mandibular premolars), caution should be exercised when interpreting these results.

## 5. Discussion

This study, which evaluated 118 teeth restored with radicular posts, provides significant insights into the factors influencing periapical health following combined endodontic and prosthetic treatment. The final sample, derived from an initial pool of 295 teeth, underscores the importance of strict inclusion criteria to ensure reliable radiographic assessment. The overall prevalence of periapical lesions (PAI scores 3–5) in our study was 31.4%, a finding that aligns closely with the findings of Almohefer et al. [13], who reported a 35.9% rate of periapical lesions in a comparable radiographic assessment. This consistency confirms that periapical pathology remains a notable clinical challenge, even after comprehensive treatment.

The present findings are consistent with previous epidemiological studies on post-restored teeth. Farah et al. [7] reported a prevalence of apical periodontitis of 22%, with higher rates observed in teeth with inadequate root canal fillings (38%) compared with adequately treated roots (14%). Although the overall prevalence in the present study was higher (31.4%), this difference may be attributed to variations in study design, radiographic techniques, and population characteristics.

This present investigation yielded two paramount, highly significant factors directly impacting periapical status. First, the density of the root canal filling was significantly associated with periapical status, with a higher prevalence of periapical lesions observed in teeth with inadequate density (44.1%) compared with those with satisfactory density (14%) (*p* < 0.001). This finding is consistent with Zhang et al. [14], who identified filling density, as assessed by CBCT, as an important factor in periapical healing. Radiographically inadequate density may indicate voids and unfilled canal spaces, which can serve as reservoirs for microbial persistence and subsequent failure [15].

However, it should be noted that this parameter primarily reflects the quality of the initial endodontic treatment rather than the effect of the post-and-core restoration itself.

Therefore, the observed association should be interpreted with caution, as it may be related to pre-existing endodontic conditions rather than the restorative procedure under investigation.

Second, the integrity of the coronal seal was decisively implicated by the significant association (*p* = 0.008) between a gap at the post-core/obturation interface and periapical disease. The prevalence of lesions was more than double in teeth with a gap (44.9%) compared with those without (21.7%). This finding provides clear radiographic evidence that a defect at this junction compromises the apical seal, likely through mechanisms of microleakage. As theorized by Geramipanah et al. [16], thermomechanical cycling can induce micro-movements, leading to microscopic gaps that permit the ingress of bacteria and toxins. Consequently, the present results strongly advocate for the verification of post fit with a control radiograph prior to final cementation and support the use of customized or anatomical posts to minimize such critical discrepancies.

In contrast to these strong associations, these results find no statistically significant link (*p* = 0.550) between the length of the remaining apical filling (using a 4 mm threshold) and periapical health. Although a non-significant trend towards better outcomes was observed with an adequate seal length (29.6% vs. 35.1% lesions), the lack of statistical significance is instructive. It suggests that a root canal filling of technically acceptable length cannot guarantee success if its density is compromised or if the coronal seal is defective. This observation refocuses clinical priority from a single linear metric to the overall quality of the seal and is consistent with the perspective of Alotaibi et al. [17], who emphasize a holistic assessment of the endodontic treatment.

A particularly suggestive finding was the relationship between systemic health and periapical status. While the result did not cross the conventional threshold of significance (*p* = 0.056), the clinical relevance is considerable: the prevalence of lesions was 66.7% in patients with systemic conditions versus 29.5% in healthy patients. This strong trend, likely limited by the small subgroup of systemically compromised patients (*n* = 6), acts as a critical alert. It aligns with large-scale studies that have established significant correlations between systemic diseases like diabetes and impaired periapical healing [18,19]. The biological plausibility is strong, as comorbidities can alter immune responses and compromise local vascularization, thereby hindering the resolution of apical periodontitis [20].

The analysis by tooth type and arch revealed interesting variations, with the highest prevalence observed in maxillary premolars (33.3%) and incisors (32.1%). The overrepresentation of maxillary teeth (83.9% of the sample) and the underrepresentation of mandibular molars are limitations that warrant caution in interpreting these rates. However, the generally higher prevalence in the maxilla is consistent with previous findings, which have similarly reported a predominance of periapical lesions in this arch. This trend may be attributed to factors such as trauma and esthetic treatment demands, as suggested by Alotaibi et al. [21]. The fact that maxillary incisors alone accounted for nearly half (48.6%) of all detected lesions highlights them as a critical focus for clinical vigilance. Furthermore, the absence of any association between periapical health and patient sex or age strengthens the argument that the technical quality of treatment is a more direct predictor of outcome than these demographic variables.

Several limitations must be acknowledged. The cross-sectional design precludes the establishment of causality. The retrospective aspect of the study did not allow for the inclusion of several important clinical variables, such as coronal restoration quality, type of post cement, treatment technique, and procedural errors, as these parameters were not consistently documented in the patients’ records.

A further limitation of this study is that some patients contributed more than one tooth, which may have introduced within-patient clustering. Since the analysis was performed at the tooth level without adjustment for intra-patient correlation, standard errors may have been underestimated. Future studies should consider hierarchical models or generalized estimating equations to account for this dependency structure.

The reliance on 2D periapical radiographs, while standard, is less sensitive than CBCT for detecting incipient lesions [22]. The modest sample size, particularly for certain tooth subgroups and systemically compromised patients, limited the statistical power for some comparisons. Finally, the absence of data on iatrogenic mishaps or the quality of the coronal restoration are confounding factors not controlled for in this analysis.

## 6. Conclusions

This study confirms that the long-term periapical health of endodontically treated teeth restored with radicular posts is fundamentally dependent on the quality of the endodontic and restorative seal. The absence of a gap between the post and the root canal filling, combined with a homogeneous, three-dimensional obturation density, emerged as the key protective factor against the development of periapical lesions. These findings advocate for a rigorous clinical approach that prioritizes meticulous endodontic techniques and perfect post adaptation over merely achieving an acceptable filling length.

For clinical practice, this translates to the systematic use of condensation techniques that ensure high filling density and the verification of post fit prior to cementation, potentially leveraging anatomical posts for a superior seal. A multidisciplinary strategy integrating endodontics and prosthodontics is paramount.

Future research should be directed toward longitudinal studies incorporating 3D imaging (CBCT) and standardized clinical parameters to further validate these associations. Exploring the impact of emerging technologies, such as bioactive sealers and finite element analysis, on these critical interfaces will be essential for developing refined, evidence-based protocols that optimize long-term outcomes.

## Figures and Tables

**Table 1:** Distribution of teeth according to the Periapical Index of Ørstavik.

PAI Score	Sample Size (*n*)	Percentage (%)
***1***	52	44.1
***2***	29	24.6
***3***	11	9.3
***4***	25	21.2
***5***	1	0.8

**Table 2:** Association between periapical status, post-obturation gap presence, root canal filling quality (density), and length of the remaining filling.

Variables		Periapical Lesions Present *n* (%)	Periapical Lesions Absent *n* (%)	*p*-Value
**Post-obturation gap**	Presence *N* = 49	22 (44.9%)	27 (55.1%)	*p* = 0.008
	Absence *N* = 69	15 (21.7%)	54 (78.3%)	
**Quality of root canal obturation**	Satisfactory (dense) *N* = 50	7 (14%)	43 (86%)	*p* = 0.001
	Unsatisfactory (non-dense) *N* = 68	30 (44,1%)	38 (55.9%)	
**Length of the remaining filling**	<4 mm *N* = 37	13 (35.1%)	24 (64.8%)	*p*= 0.550
	>4 mm *N* = 81	24 (29.6%)	57 (70.37%)	

**Table 3:** Distribution of Periapical Index (PAI) scores by tooth type.

Arch	Tooth Type	Lesion		Total	Percentage (%)
		Present (*n*)	Absent (*n*)		
**Mandible**	Canine (cmand)	1	2	3	33.3%
	Molar (mmand)	1	2	3	33.3%
	Premolar (pmand)	3	10	13	23.1%
**Mandible Subtotal**		5	14	19	26.3%
**Maxilla**	Canine (cmax)	4	9	13	30.8%
	Incisor (icmax)	18	38	56	32.1%
	Molar (mmax)	2	4	6	33.3%
	Premolar (pmax)	8	16	24	33.3%
**Maxilla Subtotal**		32	67	99	32.3%
**Total**		37	81	118	31.4%

## Data Availability

The data supporting the findings of this study are available from the corresponding author upon reasonable request. The data are not publicly available due to privacy and ethical restrictions.
